# Is greater generic competition also linked to lower drug prices in South Korea?

**DOI:** 10.1186/s13561-020-00289-6

**Published:** 2020-09-15

**Authors:** Kyung-Bok Son

**Affiliations:** College of Pharmacy, Ewha Womans University, 52Ewhayeodae-gil, Seodaemun-gu, Seoul, 03760 South Korea

**Keywords:** Generics, Competition, Drug price, Market, South Korea

## Abstract

**Background:**

Although the association between the price of generic drugs and market competitiveness has been explored in various high-income countries, this association has not been empirically evaluated in South Korea. We aim to determine the association between the prices of generic drugs and market competitiveness in South Korea.

**Methods:**

A list of originator drugs approved by the national authority from 2000 to 2019 and their corresponding generic drugs were grouped along with the baseline information. The market was categorized into four groups based on the number of manufacturers: duopoly (2 manufacturers); low- (3–25 manufacturers); medium- (26–75 manufacturers); and high-competition (more than 76 manufacturers) markets. Price variance, calculated as the difference between the maximum price and minimum price divided by the maximum price, was obtained. A multivariate regression model was applied to regress price variance on the characteristics of market competitiveness, controlling for the characteristics of the originator drugs and their price level in the market.

**Results:**

A total of 986 originator drugs were identified and then divided into duopoly (31%), low- (56%), medium- (9%), and high-competition (4%) markets; the median of the price variance for these markets was 0.013, 0.077, 0.200, and 0.228, respectively. In a multivariate regression model, price variance was associated with the characteristics of the originator drug, including the Anatomical Therapeutic Chemical classification, the route of administration, and the approval year. Controlling for the characteristics of the originator drugs, market competitiveness was positively associated with price variance.

**Conclusions:**

The positive association between price variance and market competitiveness is still consistent in South Korea, where rare price competition among a large number of generic manufacturers has been reported. However, no significant price variance was observed between medium- and high-competition markets. These findings support policies for managing a large number of generic manufacturers in South Korea.

## Background

When a pharmaceutical patent or statutory exclusivity expires, manufacturers can be granted marketing authorization for generic substitutes and penetrate originator markets [1–4]. In principle, generic drugs contain exactly the same active ingredient as the originator drugs, and generic drugs are certified by authorities to be perfect substitutes for the originator [5–8]. Thus, the entry of a perfect substitute, or a generic drug, will trigger price competition, bringing to an end the monopoly rent enjoyed by the originator manufacturer [9–13].

Price competition in pharmaceuticals, particularly for prescription drugs, matters in high-income countries [14–17]. Prescription drug expenditures in the United States have increased at a pace far beyond that in other sectors of the health system [18–20]. More specifically, manufacturers of originator drugs set prices as high as the market will bear during the limited period of patent or statutory exclusivity. To address these issues, governments have established several ways to negotiate lower prices for originator drugs and to expand the use of health technology assessment for reimbursement decisions [21–23]. Furthermore, governments are assessing how they can lower pharmaceutical expenditures through generics [14, 24].

Generic drugs can be used for discounted price compared to the originate drug. The role of generic drugs or the number of generic manufacturers in reducing drug prices has been well documented in the previous literature [11, 12]. The price of generics with 2 or fewer manufacturers is more likely to be increased compared to that of generics with 3 or more manufacturers. Sometimes, price hikes occur for generic drugs in markets where an insufficient number of generic manufacturers exist. The price of pyrimethamine, albendazole, and praziquantel increased by 5433%, 1920, and 356%, respectively [15, 25, 26]. These cases demonstrate that the number of generic manufacturers and the introduction of generics are critical in managing drug prices and pharmaceutical expenditures.

Although the association between the price of generic drugs and market competitiveness has been explored in various high-income countries, this association has not been empirically evaluated in South Korea. The pharmaceutical market in South Korea is characterized by a large number of generic manufacturers, specifically 416 manufacturers, with rare price competition among generics [16, 27]. Thus, South Korea is an ideal case to test the association between the price of generics and market competitiveness. In the present study, we used a whole list of reimbursed medicines under the National Health Insurance Services (NHIS) to investigate the association between competition and lower drug prices in South Korea.

## Methods

This study is interested in investigating the association between the price of generics and market competitiveness in South Korea. To this end, a list of originator drugs approved by the national authority from 2000 to 2019 and their corresponding generic drugs were grouped along with the baseline information. We compiled the originators and their corresponding generics as the subjects of the study, counted the number of available drugs, and retrieved their price under the NHIS. In this study, an originator is defined as a drug that was the first to be granted marketing authorization, while generics are defined as drugs that have the same active ingredient, strength, and route of administration as the originator and that were granted marketing authorization after the originator.

### Data sources

The list of reimbursed medicines under the NHIS, provided by the Health Insurance Review and Assessment Services (HIRA), was retrieved. The list contains information on drugs such as the generic and proprietary name of the drug and its strength, route of administration, manufacturer, and reimbursed price. Information on all drugs approved by the Ministry of Food and Drug Safety (MFDS) from 2000 to 2019 was also extracted. In particular, the Korea Pharmaceutical Information Service (KPIS)^1^ provides information on drugs such as the generic and proprietary name of the drug and its strength, route of administration, Anatomical Therapeutic Chemical (ATC) classification, type (including chemicals and biologics), date of marketing approval, and manufacturer. With the information on the generic name of the drug and its strength and route of administration, the list of originator drugs and their corresponding generics were grouped.

### Study design

#### Dependent variables

We used price variance, calculated as the difference between the maximum price and minimum price divided by the maximum price in the originator-generic set, as the dependent variable to measure the price competition.

#### Control variables

We chose a set of variables, specifically the characteristics of the originator drug and its manufacturer, to control for their effects. First, we categorized the characteristics of the originator drug based on the drug’s ATC classification, route of administration, year of marketing authorization, and type. Second, we grouped the manufacturers of the originator drugs into domestic and overseas manufacturers based on the origin of the manufacturers. In particular, the dataset provided by the Ministry of Trade, Industry and Energy was used to identify the origin of the manufacturers.^2^

#### Independent variables

The characteristics of the market were used to determine the association between the price of generics and market competitiveness. First, we counted the manufacturers of a certain drug, including both the originator and generics, and categorized the market into four groups based on the cumulative number of manufacturers: duopoly, low-, medium-, and high-competition markets. In a duopoly market, only two manufacturers exist: one is the originator manufacture, and the other is the generic manufacturer. A low-competition market is defined as a market with more than two and less than 26 manufacturers, while a medium-competition market is defined as a market with more than 25 and less than 76 manufacturers. Similarly, a high-competition market is defined as a market with more than 75 manufacturers participating in the market. Second, we gathered information on the maximum price in the set to understand the characteristics of the drug in the market and categorized the maximum unit price into five levels. Level I indicates low-cost medicine that is under 1000 KRW (approximately 0.86 USD), while Level V indicates high-cost medicine, which is above 1 million KRW (approximately 864 USD). Finally, we retrieved information regarding the designation of new drug applications by the MFDS.

### Statistical analysis

Descriptive analyses were used to present the differences between the four markets, including the duopoly, low-, medium-, and high-competition markets. In particular, the chi-squared test was applied for the categorical variables, and analysis of variance was conducted for the continuous variables to examine whether the variables of interest differed across the four markets.

A multivariate regression model was applied to examine the factors that affected the price variance, particularly for market competitiveness. The variables were the characteristics of the originator drug, manufacturers, and the market. In a simple model, the characteristics of the originator drug and manufacturers were included. Then, we added the market variables (new drug designation, market competitiveness, and maximum reimbursed price) in the expanded model. We used heteroscedasticity-consistent standard errors for the analysis to obtain the correct standard error against heteroscedasticity. Data management and analysis were performed using R statistical software (version 3.4.3). Statistical significance is noted by *p*-values less than 0.05.

$$ {\mathrm{Price}\ \mathrm{variance}}_{\mathrm{i}}={\beta}_0+{\beta}_1{\mathrm{ATC}}_{\mathrm{i}}+{\beta}_2{\mathrm{Route}}_{\mathrm{i}}+{\beta}_3{\mathrm{Year}}_{\mathrm{i}}+{\beta}_4{\mathrm{Types}}_{\mathrm{i}}+{\beta}_5{\mathrm{Manufacturer}}_{\mathrm{i}}+{\varepsilon}_{\mathrm{i}} $$

$$ {\mathrm{Price}\ \mathrm{variance}}_{\mathrm{i}}={\beta}_0+{\beta}_1{\mathrm{ATC}}_{\mathrm{i}}+{\beta}_2{\mathrm{Route}}_{\mathrm{i}}+{\beta}_3{\mathrm{Year}}_{\mathrm{i}}+{\beta}_4{\mathrm{Types}}_{\mathrm{i}}+{\beta}_5{\mathrm{Manufacturer}}_{\mathrm{i}}+{\beta}_6{\mathrm{Competitiveness}}_{\mathrm{i}}+{\beta}_7{\mathrm{Price}}_{\mathrm{i}}+{\beta}_8{\mathrm{NDA}}_{\mathrm{i}}+{\varepsilon}_{\mathrm{i}} $$

## Results

### Subjects of the study

**Fig. 1:**
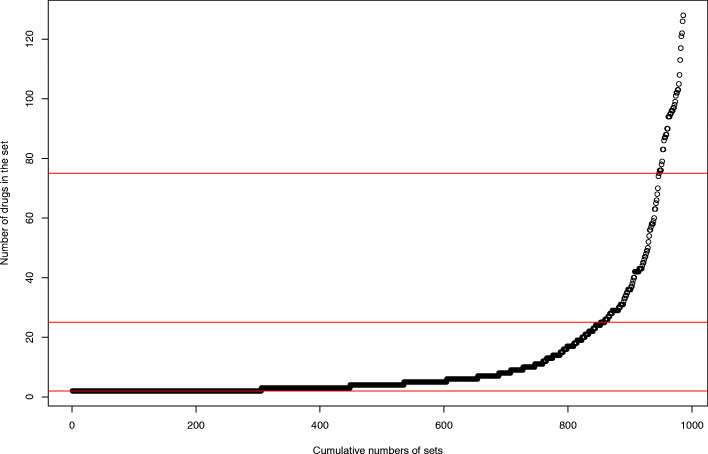
The cumulative number of sets and the number of drugs in the set

During a 20-year period, a total of 986 sets of originator and corresponding generic drugs were identified. Note that a monopoly market, in which generic drugs are not available, was excluded from our model. Figure 1 presents the cumulative number of sets and the number of drugs in the set, indicating that one-third of sets are duopoly markets. Interestingly, 39 sets (4%) include more than 75 generic manufacturers.

### Descriptive analysis

Table 1 presents the descriptive statistics of the dependent and explanatory variables in our model. The subjects were categorized into four markets based on the cumulative number of manufacturers in the market. Approximately 31, 56, 9, and 4% of the markets are duopoly, low-, medium-, and high-competition markets, respectively. The mean and median of price variance among all sets were 0.1167 and 0.0714, respectively, indicating that generics are available in the market discounted by 11.67 and 7.14%. When we separated the markets, increased trends were observed in the mean and median of price variance. The mean of price variance was 0.0683, 0.1160, 0.2268, and 0.2566 for duopoly, low-, medium-, and high-competition markets, respectively. Similarly, the median of price variance for the same markets was 0.0130, 0.0774, 0.2004, and 0.2286.

**Table 1:** Characteristics of the subjects in this study

Variables	All	Duopoly (2)	Low-competition (3–25)	Medium- Competition (26–75)	High-competition (76-)	*p*-value
	*N* = 986	305	554	88	39	
Price variance						
Mean	0.1167	0.0683	0.1160	0.2268	0.2566	< 0.0001
Median	0.0714	0.0130	0.0774	0.2004	0.2286	
SD	0.1416	0.1000	0.1366	0.1782	0.1693	
ATC						
1 (J/L)	168	58	97	11	2	< 0.0001
2 (A/B/C)	302	100	140	41	21	
3 (M/N)	239	57	148	24	10	
4 (Others)	277	90	169	12	6	
Route						
Oral	635	156	354	86	39	< 0.0001
Injection	206	103	101	2	–	
Others	145	46	99	–	–	
Year						
Mean	2007	2008	2007	2006	2005	0.0001
Median	2006	2007	2006	2006	2005	
SD	5.3662	5.2816	5.5529	4.5851	3.8231	
Types						
Biologics	50	28	9	9	4	< 0.0001
Chemicals	936	277	545	79	35	
New drug application						
Yes	154	34	96	13	11	0.0142
No	832	271	458	75	28	
Manufacturers						
Domestic	623	187	372	49	15	0.0008
Overseas	363	118	182	39	24	
Price						
I (< 10^3^ KRW)	502	120	283	67	32	< 0.0001
II (10^3^ ≤ < 10^4^)	269	86	159	17	7	
III (10^4^ ≤ < 10^5^)	154	67	83	4	–	
IV (10^5^ ≤ < 10^6^)	55	26	29	–	–	
V (10^6^≤)	6	6	–	–	–	

**Fig. 2:**
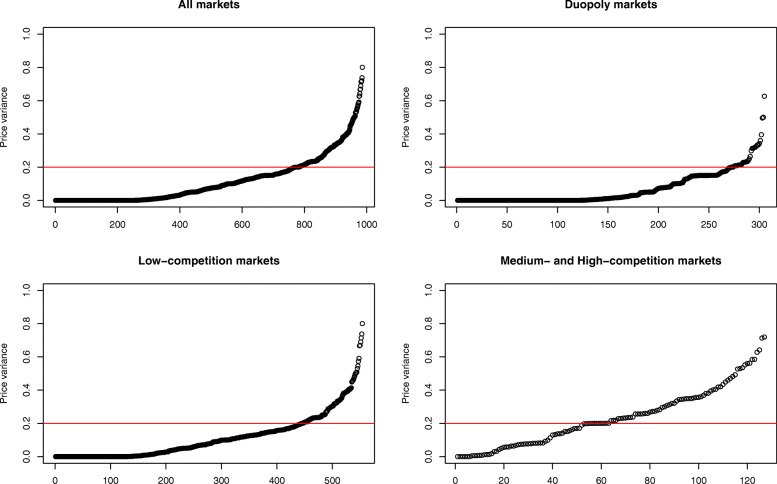
The cumulative number of sets and their price variance sorted by market types

In a similar vein, Fig. 2 presents the cumulative number of sets and their price variance. The first graph in Fig. 2 shows the curve for the whole market, while the remaining graphs present the curve for the duopoly, low-, and medium- and high-competition markets. The majority of sets in duopoly and low-competition markets present a small price variance, while a large number of sets in medium- and high-competitive markets present a large price variance. More specifically, 89, 80, 45, and 41% of sets in the duopoly, low-, medium-, and high-competition markets, respectively, show a price variance less than 0.2. Interestingly, the difference between the duopoly and low-competition markets (89% versus 80%) and the difference between the medium- and high-competition markets (45% versus 41%) were marginal.

The characteristics of the originator drug, its manufacturer, and the market are also presented in Table 1. Significant differences were noted among the four markets. In particular, the ATC classification, route of administration, year of marketing authorization, type of originator, designation of new drug applications, characteristics of the manufacturer, and reimbursed price were different among four markets. Note that 39 out of 986 sets (4%) include more than 75 generic drugs. The majority of sets in the high-competition market were chemical drugs (90%), belonging to the alimentary tract and metabolism (A), blood and blood forming organs (B), and the cardiovascular system (C) in the first level of the ATC classification (54%). Additionally, all sets in the high-competition market were oral forms, including tablets and capsules, and their price was less than 10,000 KRW. These observations imply that low-priced chemical drugs used to treat chronic disease were more likely to be included in the high-competition market.

### A regression analysis

Table 2 presents a multivariate regression for the price variance in the 986 sets. Note that price variance was calculated by the difference between the maximum price and minimum price divided by the maximum price in the sets. Thus, increased price variance indicates price competition in the set. Two models, including the simple model and the expanded model, are presented in Table 2.

**Table 2:** Adjusted association between generic competition and drug prices

Variables	The Simple Model			The Expanded Model		
	Estimates	Standard Error	*P*-value	Estimates	Standard Error	*P*-value
ATC (reference J/L)						
2 (A/B/C)	−0.0193	0.0131	0.1409	0.0017	0.0133	0.8974
3 (M/N)	0.0220	0.0149	0.1415	0.0395	0.0156	0.0114
4 (Others)	−0.0498	0.0151	0.0010	−0.0254	0.0150	0.0904
Route (reference Oral)						
Injection	−0.0342	0.0117	0.0035	−0.0309	0.0138	0.0255
Others	−0.0444	0.0133	0.0009	−0.0337	0.0139	0.0153
Year	−0.0066	0.0007	< 0.0001	−0.0045	0.0007	< 0.0001
Types (reference Chemicals)						
Biologics	−0.0175	0.0186	0.3475	−0.0457	0.0180	0.0114
Market (reference Duopoly)						
2 (3–25)				0.0396	0.0080	< 0.0001
3 (26–75)				0.1473	0.0198	< 0.0001
4 (76 -)				0.1650	0.0259	< 0.0001
New drug application (reference No)						
Yes				0.0430	0.0141	0.0024
Manufacturers (reference Domestic)						
Overseas				0.0087	0.0100	0.3836
Price (reference < 10^3^ KRW)						
II (10^3^ ≤ < 10^4^)				0.0418	0.0108	0.0001
III (10^4^ ≤ < 10^5^)				0.0422	0.0158	0.0076
IV (10^5^ ≤ < 10^6^)				0.0799	0.0271	0.0033
V (10^6^≤)				0.1023	0.0371	0.0060

The route of administration and the approval year of the originator were significantly associated with price competition in the simple model. More specifically, drugs in injection and other forms were less likely to experience price competition than drugs in oral forms. In a similar vein, drugs approved recently were less likely to experience price competition. However, the type of originator (chemicals or biologics) was not significantly related to price competition.

In the expanded model, we observed consistent results for the variables on the route of administration and approval year. However, the type of originator was significantly related to price competition in the expanded model. Furthermore, market competitiveness and the price of the originator were significantly related to price variance. Drugs in the low-, medium-, and high-competition markets were more likely to experience price competition than drugs in the duopoly market. Similarly, drugs in Levels II, III, and IV (high-cost drugs) were more likely to experience price competition than drugs in Level I (low-cost drugs). Finally, the designation of new drug applications (NDAs) was also significantly related to price competition.

## Discussion

Governments and payers believe that there should be opportunities for cost savings in drug markets [14, 28, 29]. Based on this belief, governments have applied measures to introduce generic drugs in a timely manner, to stimulate price competition among generic drugs, and to increase generic penetration in various clinical settings. Meanwhile, several studies provide insights into competition, particularly the number of manufacturers, and its effect on lowering drug prices [11, 12, 26].

In the previous literature, the number of generic manufacturers for specific blockbuster markets in South Korea has been reported, with conclusions that the South Korean market is extraordinary compared to the number of generic manufacturers in other high-income countries and rare price competition among a large number of generic manufacturers [16, 27]. We aimed to test the association between competition among manufacturers and drug prices in South Korea. To this end, a list of originator drugs approved by the national authority from 2000 to 2019 and their corresponding generic drugs were grouped along with the baseline information.

### Number of generic manufacturers in South Korea

A total of 986 sets of originators and their corresponding generic drugs were identified in this study. Among them, 127 sets (13%) include more than 25 generic manufacturers. Furthermore, 39 sets (4%) include more than 75 generic manufacturers. These results are surprising when we directly compare the number of generic manufacturers in South Korea with the number in the United States. Li et al. (2018) categorized the topical dermatologic generic drug market in the United States into four groups [12]: 1–2, 3–4, 5–6, and more than 6 generic manufacturers. Similarly, Alpern et al. (2017) provide information on the number of oral antibiotic drug manufacturers in the United States [26]. In their study, the number of manufacturers for cefuroxime 250 mg was 9, and this number was the maximum among other oral antibiotic drugs. For the same drug, however, the number of manufacturers in South Korea is 38. This situation is even more surprising given that the pharmaceutical market in South Korea represents only 1.5% of the global market [30].

It is also noteworthy that 305 sets (31%) are in the duopoly market. In this market, the mean and median of price variance were 0.0683 and 0.0130, respectively, indicating that generics discounted by 6.83 and 1.30% are available. These figures, particularly the median (0.0130) of price variance, imply scarce price competition in the duopoly market. Furthermore, a number of expensive drugs (10.5%), which are above 100,000 KRW, belong to the duopoly market. Thus, policies to encourage marketing competition in duopoly markets should be formulated. In particular, off-patent drugs with few manufacturers should be prioritized areas to be addressed, and the MFDS’s role in marketing authorizations for generics, including a timely review process for generic drug applications in the duopoly market, should be established.

### Association between competition and generic prices

Given the characteristics of the South Korean market, we categorized the groups into four markets: duopoly, low-, medium-, and high-competition markets. In the descriptive analysis, we found that the majority of sets in duopoly markets present a small price variance, while a large number of sets in high-competitive markets present a large price variance. In the regression model, we found a positive association between price variance and the number of generic manufacturers, controlling for the ATC classification, route of administration, and type of originator drug. These observations are consistent with the experiences in other countries. It was well documented that market competition levels were associated with a price change in generic drugs in the United States. However, in interpreting these observations, the differences in health systems between the United States and South Korea should be noted.

The United States has a market-based health system. Approximately half of health care spending is publicly funded, but the beneficiaries of the funded program obtain health care services via private markets [31, 32]. In the pharmaceutical sector, tighter pricing and reimbursement schemes do not exist, not even for generic drugs, and few generic manufacturers participate in the market [18]. On the other hand, the South Korean health system is financed by the National Health Insurance Services to cover the entire population [33]. In the pharmaceutical sector, there is a positive list system in which only drugs included in the formulary that demonstrate cost-effectiveness can be reimbursed [23]. Furthermore, tighter pricing and reimbursement schemes exist for generic drugs as well as new medicines, and a number of manufacturers participate in the market.

Given these differences, the United States is interested in a price increase for a certain drug. Specifically, price hikes have occurred for generic drugs on the market where an insufficient number of generic manufacturers exist. Thus, researchers are more interested in price increases in generics for which few manufacturers exist. On the other hand, many manufacturers produce generic drugs in South Korea. It is reasonable to anticipate fierce price competition among a large number of generic drugs. However, it has been reported that the increased number of generic manufacturers have not triggered price competition in reality [16, 23]. Thus, we are interested in price variance among a large number of originator-generic sets.

This study demonstrates the association between the price of generics and market competitiveness in South Korea. However, in our observations, the association was not linear: the difference in the mean and median of price variance between the medium- and high competition markets was marginal (0.0298 and 0.0282, respectively) in Table 1. Furthermore, the estimates for the medium- and high competition markets were similar (0.1473 and 0.1650, respectively) in Table 2. These findings suggest that policies for managing the number of generic manufacturers fewer than 76 manufacturers could be introduced in the market without limiting price competition.

### Limitations

This study includes the entire list of originator drugs approved by the national authority from 2000 to 2019 and their corresponding generic drugs. Thus, the study findings might be generalizable to all types of drugs in the South Korean market. However, our study has limitations. While we attempted to capture all drugs approved from 2000 to 2019, information on their sales was not included. In a similar vein, this study used price variance, calculated as the difference between the maximum price and minimum price in the sets divided by the maximum price, to measure price competition. However, the market share of the minimum priced generic drug might be marginal, implying that the effect of price competition might have less implications in managing pharmaceutical expenditure.

## Conclusions

The positive association between price variance and market competitiveness is still consistent in South Korea, where rare price competition among a large number of generic manufacturers has been reported. However, no significant price variance between medium- and high-competition markets was observed. These findings suggest that policies for managing the number of generic manufacturers fewer than 76 manufacturers could be introduced in the market without limiting price competition. Furthermore, it should be noted that the majority of drugs are in a duopoly market without price competition in South Korea. Thus, policies to encourage marketing competition and to address rare price competition issues should be formulated for the duopoly market. In particular, off-patent drugs with few manufacturers should be prioritized areas to be addressed, and the MFDS’s role in timely marketing authorizations for generics should be established.

## Data Availability

We used publicly available data, provided by the Health Insurance Review and Assessment Services and the Ministry of Food and Drug Safety.

## Abbreviations

ATC: Anatomical Therapeutic Chemical
HIRA: Health Insurance Review and Assessment Services
KPIS: Korea Pharmaceutical Information Service
MFDS: Ministry of Food and Drug Safety
NDA: New drug applications
NHIS: National Health Insurance Services

## Footnotes

1 Available at https://biz.kpis.or.kr/kpis_biz/index.jsp

2 Available at http://www.motie.go.kr/motie/py/sa/companyGuide/companyguide.jsp
